# Arthropods Under Pressure: Stress Responses and Immunity at the Pathogen-Vector Interface

**DOI:** 10.3389/fimmu.2020.629777

**Published:** 2021-02-15

**Authors:** Kristin L. Rosche, Lindsay C. Sidak-Loftis, Joanna Hurtado, Elizabeth A. Fisk, Dana K. Shaw

**Affiliations:** Program in Vector-borne Disease, Department of Veterinary Microbiology and Pathology, Washington State University, Pullman, WA, United States

**Keywords:** vector-borne diseases, vector competence, vector-borne pathogens, arthropod immunity, eukaryotic stress response, integrated stress response, unfolded protein response

## Abstract

Understanding what influences the ability of some arthropods to harbor and transmit pathogens may be key for controlling the spread of vector-borne diseases. Arthropod immunity has a central role in dictating vector competence for pathogen acquisition and transmission. Microbial infection elicits immune responses and imparts stress on the host by causing physical damage and nutrient deprivation, which triggers evolutionarily conserved stress response pathways aimed at restoring cellular homeostasis. Recent studies increasingly recognize that eukaryotic stress responses and innate immunity are closely intertwined. Herein, we describe two well-characterized and evolutionarily conserved mechanisms, the Unfolded Protein Response (UPR) and the Integrated Stress Response (ISR), and examine evidence that these stress responses impact immune signaling. We then describe how multiple pathogens, including vector-borne microbes, interface with stress responses in mammals. Owing to the well-conserved nature of the UPR and ISR, we speculate that similar mechanisms may be occurring in arthropod vectors and ultimately impacting vector competence. We conclude this Perspective by positing that novel insights into vector competence will emerge when considering that stress-signaling pathways may be influencing the arthropod immune network.

## Introduction

Among arthropods, the adaptation to blood-feeding is a life history trait that evolved independently at least 20 times (1). From the arthropod’s perspective, a hematophageous lifestyle has both benefits and drawbacks. Blood is a good source of proteins and lipids, which are necessary for development and egg production (2–4). However, blood-feeding comes with a variety of risks and stressors (5) including long periods between nutrient supplementation (6–13), thermal stress associated with the influx of a hot blood meal (5, 14–16), heme toxicity (17–30) and excess amounts of ions and water (31). Cells respond to acute environmental changes by activating stress responses that temporarily increase tolerance limits in adverse conditions and/or eliminate stressful stimuli. Being able to respond to stressful stimuli is an evolutionary advantage, which explains the highly conserved nature of cellular stress responses across eukaryotes (32–40).

For arthropods that transmit disease, another stressor is the presence of pathogens with incoming blood meals (41–44). Although vector-borne pathogens do not typically kill their arthropod vectors (45), infection does impart stress on the host by parasitizing nutrients, secreting toxic by-products and/or causing physical damage (46). For this reason, arthropod immune processes responding to infection are a key factor influencing vector competence (47–53). From mammals, it is now recognized that innate immunity is tightly intertwined with cellular stress responses and may represent an ancient mode of host defense against infection (32–34, 54–60). Whether stress-responses also intersect with arthropod immunity and how this may influence vector competence of blood-feeding arthropods is not known. Herein we briefly outline current knowledge of two well-characterized cellular stress response mechanisms, the Unfolded Protein Response (UPR) and the Integrated Stress Response (ISR) and discuss evidence that stress signaling impacts immunity. We then cite examples of cellular stress responses mediating outcomes at the host-pathogen interface in mammals and conclude that, given the well-conserved nature of the UPR and the ISR, similar crosstalk may be occurring in arthropods that would fundamentally impact vector competence.

## Arthropod Innate Immune Signaling

Arthropod innate immune pathways are best characterized in the model organism *Drosophila* which are briefly summarized owing to space constraints. The Toll pathway is generally characterized as responding to Gram-positive bacteria and fungi, resulting in activation of Rel-family transcription factors Dorsal and Dif (Dorsal-related immunity factor) (61–64). The IMD (immune deficiency) pathway is analogous to the tumor necrosis factor receptor (TNFR) pathway in mammals (65) and is initiated by Gram-negative bacteria, although exceptions outside of *Drosophila* have been observed (66–71). The Janus Kinase (JAK)-signal transducers and activators of transcription (STAT) pathway, first described in mammals, is activated in the presence of bacterial or protozoan pathogens as well as more than 35 ligands, including interferons (IFN) and interleukins (IL) (72, 73). In arthropods, the JAK-STAT pathway is induced by the endogenous cytokine Unpaired (Upd) (74). Owing to space constraints, we refer readers to excellent reviews that comprehensively cover arthropod Toll, IMD and JAK-STAT signaling (65, 75, 76).

## The Unfolded Protein Response

**Figure 1:**
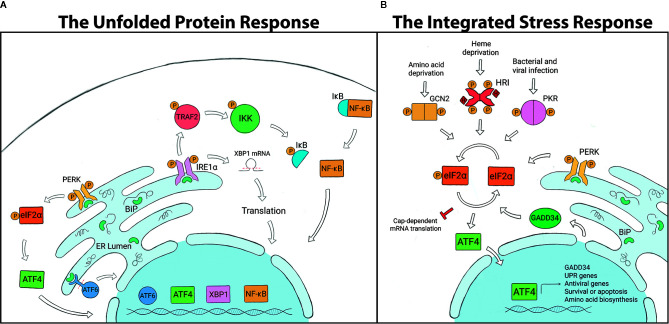
**(A)** The Unfolded Protein Response (UPR) is regulated by three transmembrane endoplasmic reticulum (ER) receptors: PERK, ATF6 and IRE1α. BiP binds to and holds the receptors in an inactive state under non-stressed conditions. The accumulation of unfolded proteins in the ER leads to the disassociation of BiP from the receptors and UPR activation. PERK phosphorylates eIF2α and induces ATF4, which controls transcription of *chop* and *gadd34*. ATF6 is cleaved and translocated to the nucleus to regulate expression of GRP94, p58IPK, and UPR-associated proteins. IRE1α splices *xbp1*, which is translated into a protein that controls genes involved in lipid biosynthesis, ER associated degradation pathway (ERAD) and chaperone production. IRE1α can also recruit TRAF2, which leads to NF-kB, ASK1 and JNK signaling. **(B)** The Integrated Stress Response (ISR) is initiated by stress-specific stimuli that activate kinases GCN2, HRI, PKR and PERK, which all converge on the regulatory molecule eIF2α. Phosphorylation of eIF2α halts global protein translation and upregulates the transcription factor ATF4, which determines cell fate. To terminate the ISR, ATF4 induces GADD34 expression which dephosphorylates eIF2α and allows global protein translation to resume.

The UPR is a cellular stress response mechanism that is highly conserved across species, from single-celled eukaryotes to mammals (**Figure 1**). The UPR is triggered when the endoplasmic reticulum (ER) is under stress, which can result from a variety of stimuli such as oxidative stress, changes in temperature or pH, lack of nutrients or infection (32–34, 77–84). Such conditions can impart stress when protein-folding requirements exceed the processing capacity of the ER, causing an accumulation of unfolded proteins in the ER lumen. The UPR is activated through any combination of 3 transmembrane receptors: PERK (PKR-like ER kinase), ATF6 (activating transcription factor 6) or IRE1α (inositol-requiring enzyme 1α). In a non-stressed state, the negative regulator, BiP (binding immunoglobulin protein; also known as GRP78), keeps all three receptors in an inactive state by binding to them. Upon activation, BiP disassociates from the receptors, thereby activating signaling. Disassociation allows IRE1α and PERK to oligomerize and autophophorylate (85–87), whereas ATF6 is released for migration to the Golgi (88, 89). If homeostasis cannot be restored, the UPR will switch from pro-survival to proapoptotic outcomes (90).

PERK is a type I transmembrane protein kinase that has dual roles in the UPR as well as the ISR (91). When activated, PERK dimerizes, autophosphorylates and then also phosphorylates the regulatory molecule, eIF2α (eukaryotic translation initiation factor 2α). Phosphorylated eIF2α promotes cell survival by temporarily inhibiting protein translation, which decreases the amount of proteins entering the ER and alleviates the demand for protein folding. While inhibiting the translation of most mRNAs, eIF2α selectively induces the expression of some proteins including ATF4 (activating transcription factor 4). ATF4 can activate transcription of the growth arrest and DNA damage-inducible protein, GADD34, which negatively regulates eIF2α phosphorylation, or CHOP (C/EBP homologous protein), which is a proapoptotic factor (85, 92, 93).

ATF6 is a type II transmembrane protein that contains a bZIP transcription factor within the cytosolic domain. Once BiP disassociates from ATF6, it is transported to the Golgi compartment by COPII-containing vesicles (33, 94, 95). ATF6 is proteolytically processed by the Golgi-resident proteases S1P and S2P (site-1/2 proteases), which cleave the amino-terminal portion and allow the bZIP transcription factor to translocate to the nucleus (81, 94, 96). ATF6 upregulates the expression of GRP94 (endoplasmin) to increase the ER’s folding capacity and p58IPK to induce the ER associated degradation pathway (ERAD). ATF6 also induces the expression of other UPR-associated proteins including BiP and XBP1 (X-box binding protein) (32, 60, 97, 98).

IRE1α is a type I transmembrane protein with a cytosolic serine/threonine kinase domain and an RNase (ribonuclease) domain. When the ER is stressed, IRE1α autophosphorylates and splices the inactive mRNA *xbp1^U^* into *xbp1^S^*, which is then translated into a protein (33, 81, 99, 100). XBP1 translocates to the nucleus where it induces genes that are involved in lipid biosynthesis, ERAD and chaperone production (100, 101). IRE1α signaling also limits the amount of new proteins entering the ER through regulated IRE1α-dependent decay (RIDD), which degrades mRNA (102). With high levels of ER stress, IRE1α recruits the adaptor protein TRAF2 (TNF receptor-associated factor 2) a component of the TNFR pathway. IRE1α-TRAF2 association recruits the IKK complex, leading to NF-κB activation and proinflammatory responses (33, 103, 104). Alternatively, IRE1α-mediated signaling through TRAF2 can also lead to ASK1 (apoptosis signal regulating kinase 1; also known as mitogen-activated protein kinase 5, MAP3K5) activation and downstream JNK (JUN N-terminal kinase) signaling to induce apoptotic outcomes (33, 100, 104, 105). Less is known about UPR mechanisms in arthropods, but genome comparisons indicate that UPR-encoding genes are well-conserved between species, which may suggest similar mechanisms of action (**Table 1**).

**Table 1:** Distribution of Unfolded Protein Response (UPR) and Integrated Stress Response (ISR) genes across arthropod vectors.

Common Name	Genus	UPR and ISR genes									
		BiP	ATF6	IRE1α	XBP1	ATF4	eIF2α	PERK	GCN2	PKR	HRI
Fruit Flies	*Drosophila*	+	+	+	+	+	+	+	+	−	−
Mosquitoes	*Culex*	+	+	+	+	+	+	+	+	−	+
	*Aedes*	+	+	+	+	+	+	+	+	−	+
	*Anopheles*	+	+	+	+	+	+	+	+	−	+
Fleas	Xenopsylla*	+	+	+	+	+	+	+	+	−	−
	*Ctenocephalides*	+	+	+	+	+	+	+	+	−	+
Lice	*Pediculus*	+	+	+	+	−	+	+	+	−	+
Triatome bugs	Triatoma*	+	+	+	+	+	+	+	+	−	+
Ticks	*Ixodes*	+	+	+	+	+	+	+	+	−	+
	Dermacentor*	+	+	+	+	−	+	+	+	−	+
	Ornithodoros*	+	+	+	+	+	+	+	+	−	+
Mites	*Leptotrombidium*	+	−	+	+	−	+	+	+	−	+

NCBI’s Basic Local Alignment Search Tool (BLAST) was used with query sequences from Homo sapiens to identify putative homologs. (+) homologs identified (–), homologous gene targets not found.

*homologs found in vector transcriptomes.

## The Integrated Stress Response

The ISR is responsible for alleviating cellular stress and restoring homeostasis in eukaryotes (93, 106–109) (**Figure 1**). In mammalian cells, the ISR can be activated by one of four stress-sensing kinases: PKR (protein kinase double-stranded RNA-dependent), GCN2 (general control nonderepressible 2), HRI (heme-regulated inhibitor) and PERK (110, 111). These serine-threonine kinases are stimulated by pathological and physiological changes in the cellular environment. GCN2, a highly conserved cytoplasmic kinase, is stimulated by UV irradiation and nutrient deprivation (e.g. amino acid, glucose) (107, 108, 112). PERK is stimulated when misfolded proteins accumulate in the ER, causing ER stress (91, 113, 114). PKR is activated primarily in response to viral infections, as well as bacterial infections and oxidative stress (109, 115–117). Unlike the other kinases, HRI is mostly expressed in erythrocytes and acts as a heme sensor that is activated by iron deficiency (118).

All four stress-sensing kinases converge on a common regulatory factor: the phosphorylation of eIF2α (106, 110). Under non-stressed conditions, protein translation is initiated when the eIF2 complex (consisting of eIF2α, eIF2β and eIF2γ) binds with GTP and Met-tRNA (initiator methionyl tRNA). The ternary complex then associates with the 40S ribosome subunit to form the 43S pre‐initiation complex that binds to the 5’ end of mRNA and scans for start codons. Upon recognition, eIF2-GTP (active state) is hydrolyzed to eIF2-GDP (inactive state) and causes a conformational change to the pre-initiation complex, halting the mRNA scanning process and allowing protein translation to begin (119–123). eIF2B, a guanine nucleotide exchange factor, is essential for recycling GDP to GTP for new rounds of protein translation. Phosphorylated eIF2α attenuates protein synthesis owing to its increased affinity for eIF2B that prevents eIF2-GDP to eIF2-GTP exchange (110, 121–123).

As previously discussed in reference to PERK signaling, ATF4 is activated downstream of eIF2α phosphorylation and can act as both a transcriptional activator and repressor of genes important for determining cell fate, including *chop* and *gadd34* (124–126). In response to prolonged ER-stress, ATF4 interacts with CHOP to generate reactive oxygen species (ROS), a key signal for mediating cell death (125). Once cellular stress is resolved, ATF4 induces GADD34 which interacts with protein phosphatase 1 (PP1) to dephosphorylate eIF2α and terminate the ISR (126).

The ISR has known roles in regulating host immunity. In response to ISR activation, cells will form cytoplasmic aggregates of untranslated mRNA and proteins termed “stress granules” that influence immune signaling. Phosphorylated eIF2α induces stress granules, which become cell signaling hubs that can intercept molecules from other pathways to modulate processes such as immunity (35, 127, 128). For example, TRAF2 is sequestered into stress granules, which ultimately suppresses NF‐κB-mediated inflammation (127). The ISR can also act as an antiviral defense mechanism (129–131). A stress granule-nucleating protein G3BP1 (Ras-GTPase-activating protein SH3 domain binding protein 1) recruits and activates PKR to suppress viral protein synthesis. It also activates innate immune responses through NF-κB and JNK pathways and promotes the expression of proinflammatory cytokines such as IL-17 (117). The arthropod ISR is less-studied when compared to mammals, but comparative genomic analyses demonstrate that many ISR genes are well-conserved in arthropod vectors (**Table 1**).

## Interspecies Interactions

### UPR-Pathogen Interface

The UPR is increasingly implicated in host defense against infection (132–137). For example, mammalian macrophages limit methicillin resistant *Staphylococcus aureus* (MRSA) through IRE1α with sustained ROS production and concentrated delivery of ROS to bacteria-containing phagosomes (138). The foodborne pathogen *Camplyobacter jejuni* activates eIF2α and CHOP, while decreasing expression of *perk*, *ire1α* and *atf6* in human intestinal epithelial cells. Pharmacologically inducing the UPR prior to infection resulted in decreased *C. jejuni* invasion (139), highlighting the UPR as a host defense strategy.

Some pathogens manipulate or selectively induce the UPR to promote growth and survival. *Legionella pneumophila*, causative agent of Legionnaires’ disease, inhibits UPR-induced apoptosis with secreted effector proteins Lgt1 and Lgt2 that block IRE1α-mediated *xbp1^U^* splicing (140, 141). The intracellular pathogen *Brucella* manipulates the UPR in mammalian cells with secreted effectors TcpB and VceC (142–144), which induce IRE1α signaling and NF-кB to potentiate pro-inflammatory responses (81, 144–147). This effector-mediated manipulation appears to benefit the bacterium, with VceC providing an advantage for long-term *in vivo* colonization (144).

Vector-borne pathogens are also reported to cause ER stress and UPR activation in mammalian hosts. *Francisella tularensis*, causative agent of Tularemia vectored by ticks (*Dermacentor* spp. and *Amblyomma americanum*) and biting insects (148–151), alters the expression of *bip*, increases IRE1α phosphorylation and ATF6 activation, but decreases PERK phosphorylation and CHOP (152). IRE1α-XBP1 signaling is reported to limit *F. tularensis in vivo* with increased pathogen burdens in the liver, spleen and lungs of *xbp1* ^-/-^ mice (153). *Orientia tsutsugamushi*, the causative agent of Scrub Typhus transmitted by trombiculid mites (chiggers), induces the UPR and ERAD in HeLa cells to benefit bacterial growth, owing to the increase in available amino acids (154). The *Ixodes scapularis*-transmitted bacterium *Anaplasma phagocytophilum* induces all the three UPR branches in THP-1 cells (155).

Due to their very nature, viral replication requires host cells for protein production, which often engages the UPR. For example, herpesviruses activate one or more of the UPR receptors, but limit downstream signaling to ensure global protein translation, including viral proteins, is not halted (156–158). Many arthropod-transmitted viruses activate one or more UPR sensors in mammalian cells as well. Bluetongue virus, transmitted by *Culicoides* spp. (biting midges), induces autophagy to benefit viral replication by activating PERK-eIF2α signaling of the UPR (159). Mosquito-transmitted viruses Chikungunya (CHIKV), Dengue (DENV) and West Nile (WNV) all activate one or more UPR sensors in mammalian cells. CHIKV activates BiP and the ATF6 and IRE1α branches of the UPR, but blocks PERK signaling by suppressing eIF2α phosphorylation through the nonstructural viral protein nsP4 (160, 161). The flaviviruses DENV and WNV induce *xbp1^U^* splicing, ATF6 proteolysis, and eIF2α phosphorylation to benefit viral propagation in mammalian cells and, in the case of WNV, to inhibit type I IFN signaling (161–164). Tick-borne encephalitis virus (TBEV) and Langat virus (LGTV) are Ixodid-transmitted flaviviruses. TBEV infection activates the IRE1α and ATF6 pathways to facilitate viral replication (165). In contrast, while LGTV infection activates the UPR, PERK signaling restricts viral load (166), highlighting the importance of the UPR as a host defense mechanism.

Much less is known about the UPR-pathogen interface in arthropod vectors. Recent work shows differential regulation of ER-resident proteins involved in ERAD in *Borrelia burgdorferi-*infected adult *I. scapularis* (167). There is also evidence that arthropod-transmitted plant pathogens manipulate the UPR in their arthropod vectors. *Candidatus* Liberibacter asiaticus (CLas), causative agent of Asian citrus greening disease, is vectored by the Asian citrus psyllid *Diaphorina citri* Kuwayama (*D. citri*). Infection of *D. citri* with CLas upregulates expression of ERAD and UPR components. A related bacterium, *Candidatus* Liberibacter solanacerarum, also upregulates *ire1α* and multiple genes involved in ERAD in its arthropod vector, the potato psyllid (*Bactericera cockerelli*) (168). Although more work is required to understand what role the UPR and ERAD-mediated protein degradation have during arthropod infection, these studies demonstrate that vector-borne pathogens are interfacing with the UPR in arthropods.

### ISR-Pathogen Interface

The ISR broadly responds to a variety of stress-inducing stimuli, including invasion and damage caused by infection. *Listeria monocytogenes*-infected mammalian cells activate PERK, ATF4, eIF2α, PKR and induce the expression of CHOP. *L. monocytogenes* secretes listeriolysin O (LLO), which is a pore-forming hemolysin required for phagosomal escape and bacterial survival. Cells treated with *L. monocytogenes* LLO resulted in a similar ISR activation phenotype as infection, indicating that this effector is partially responsible for inducing the ISR. Downstream production of type I IFN activates the ISR kinase PKR, increasing its expression and activation, and further stimulating eIF2α signaling (169). Epithelial cell infection with *Shigella flexneri* disrupts host cell membranes, causing GCN2-mediated eIF2α phosphorylation. This halts global protein translation, leading to stress granule formation and autophagy that eliminates bacteria (170).

These examples reflect the role of the ISR as a host-defense strategy against infection. In response, many pathogens have evolved methods to counteract ISR-mediated defenses. For example, the causative agent of Q fever, *Coxiella burnetii*, increases eIF2α phosphorylation in a Type IV Secretion System-dependent manner and induces ATF4 and CHOP in human macrophages. However, nuclear translocation of CHOP is blocked by *C. burnetii* to prevent ER stress-induced apoptosis (171). Coronavirus protein AcP10 and picornavirus protein AiVL promote viral protein synthesis by acting as competitive inhibitors for phosphorylated-eIF2 and eIF2B interactions (172). Excellent reviews summarizing ISR-mediated antiviral responses, including stress granules, and concurrent viral evasion strategies have been published in the past several years which readers are referred to (129, 173).

Interactions between vector-borne pathogens and ISR mechanisms have been reported, with several focusing on insect-borne viruses. In mammalian cells, the sandfly fever Sicilian phlebovirus evades PKR defense mechanisms by expressing a nonstructural protein that binds to eIF2B, blocking translation inhibition and promoting viral replication (174). Rift valley fever virus, transmitted by mosquitoes and sandflies, degrades host PKR and inhibits IFN induction (175–177). WNV inhibits PKR activation and downstream phosphorylation of eIF2α and stress granule formation (178). Zika virus likewise has evolved to evade stress granule formation in host cells by repurposing host proteins, including G3BP1, to facilitate viral replication and repress normal stress granule assembly (179, 180). Other flaviviruses such as WNV, DENV and Japanese encephalitis virus hijack or further inhibit stress granule machinery to benefit replication (181–184). These studies illustrate that vectored pathogens evade ISR signaling to facilitate replication and survival.

## Concluding Remarks

Vector-borne pathogens selectively interface with the UPR and the ISR to promote survival and infection in mammals. Given the well-conserved nature of both the UPR and the ISR between evolutionarily distant species (**Table 1**), it is reasonable to speculate that vectored microbes may also be modulating the stress responses in their arthropod vectors. Moreover, this type of manipulation may be a common survival strategy used by vector-borne pathogens to suppress host defenses and create replicative niches.

Cellular stress responses are increasingly recognized as being closely intertwined with innate immunity (32–34, 54–60). For example, there are multiple reports that mammalian Toll-like receptors (TLRs) influence the UPR and the ISR. TLR2 and TLR4 in mammalian macrophages activate the IRE1α-XBP1 axis, which leads to proinflammatory TNFα and IL-6 cytokine production (153). MRSA induces *xbp1^U^* splicing in wildtype bone marrow-derived macrophages, but not in *TLR2/4/9* ^-/-^ or *myd88* ^-/-^ mutant cells, indicating that TLR signaling is required for IRE1α activation (138). The ISR kinase PKR is activated downstream from TLR3 and TLR4, which induces type I IFN (185, 186). Whether a similar phenomenon occurs in arthropods is not known, but considering the well-conserved nature of the Toll pathway between arthropods and mammals (70, 187) it is possible that this type of crosstalk is occurring across species.

Beyond TLR signaling, overlap between the UPR and ISR with other innate immunity components has also been noted. During infection, the mammalian UPR is capable of initiating an immune response by crosstalking with the TNFR pathway (33, 56–58, 188–190). IRE1α can produce proinflammatory responses by signaling through TRAF2, a component of the TNFR pathway, recruiting the IKK complex for phosphorylation and releasing NF-κB for nuclear translocation (32–34). Arthropod immunity may similarly be influenced by the UPR, as the arthropod IMD pathway is analogous to the mammalian TNFR network. PERK was shown to interact with JAK-STAT signaling in mammalian glial cells (191) and several examples of IFN production being influenced by the ISR and UPR have been reported, which could potentially influence JAK-STAT signaling in an indirect manner (169, 175–177, 185, 186, 192). Similar crosstalk between JAK-STAT and cellular stress responses may also be occurring in arthropod vectors.

Since one of the major factors determining vector competence is arthropod immunity (47–53), it is feasible that cellular stress responses may be influencing vector competence. With this in mind, understanding how stress responses may interface with arthropod immunity and conversely how vector-borne pathogens may be inducing or manipulating cellular stress responses could be important for opening new avenues in vector-borne disease control. This knowledge could be leveraged for the future design of disease transmission-blocking strategies to reduce the global burden of vector-borne diseases.

## Data Availability Statement

Publicly available datasets were analyzed in this study. This data can be found on NCBI.

## Author Contributions

KR, LS-L, JH, and DS wrote this perspective. EF designed and constructed **Figure 1** and contributed to intellectual discussions. All authors contributed to the article and approved the submitted version.

## Funding

This work was supported by National Institute of Health grants GM008336 to JH and R21AI139772, R21AI148578 and Washington State University to DKS. The content is solely the responsibility of the authors and does not necessarily represent the official views of the National Institute of Allergy and Infectious Diseases or the National Institutes of Health.

## Conflict of Interest

The authors declare that the research was conducted in the absence of any commercial or financial relationships that could be construed as a potential conflict of interest.
